# Identifying Interacting Genetic Variations by Fish-Swarm Logic Regression

**DOI:** 10.1155/2013/574735

**Published:** 2013-08-05

**Authors:** Xuanping Zhang, Jiayin Wang, Aiyuan Yang, Chunxia Yan, Feng Zhu, Zhongmeng Zhao, Zhi Cao

**Affiliations:** ^1^Department of Computer Science and Technology, Xi'an Jiaotong University, Xi'an, Shaanxi 710049, China; ^2^The Genome Institute, Washington University, St. Louis, MO 63108, USA; ^3^College of Medicine and Forensics, Xi'an Jiaotong University Health Science Center, Xi'an, Shaanxi 710061, China; ^4^Key Laboratory of the Ministry of Health for Forensic Sciences, Xi'an Jiaotong University, Xi'an, Shaanxi 710061, China; ^5^Key Laboratory of the Ministry of Education for Environment and Genes Related to Diseases, Xi'an Jiaotong University, Xi'an, Shaanxi 710061, China; ^6^Center for Translational Medicine, The First Affiliated Hospital of Xi'an Jiaotong University College of Medicine, Xi'an, Shaanxi 710061, China

## Abstract

Understanding associations between genotypes and complex traits is a fundamental problem in human genetics. A major open problem in mapping phenotypes is that of identifying a set of interacting genetic variants, which might contribute to complex traits. Logic regression (LR) is a powerful multivariant association tool. Several LR-based approaches have been successfully applied to different datasets. However, these approaches are not adequate with regard to accuracy and efficiency. In this paper, we propose a new LR-based approach, called fish-swarm logic regression (FSLR), which improves the logic regression process by incorporating swarm optimization. In our approach, a school of fish agents are conducted in parallel. Each fish agent holds a regression model, while the school searches for better models through various preset behaviors. A swarm algorithm improves the accuracy and the efficiency by speeding up the convergence and preventing it from dropping into local optimums. We apply our approach on a real screening dataset and a series of simulation scenarios. Compared to three existing LR-based approaches, our approach outperforms them by having lower type I and type II error rates, being able to identify more preset causal sites, and performing at faster speeds.

## 1. Introduction

Understanding the genotype-phenotype association is one of the major problems in human genetics. Much effort has been devoted to mapping complex traits with one or pairwise single nucleotide polymorphisms (SNPs). These studies were mainly supported by the “common disease-common variant (CDCV)” hypothesis [1, 2], which suggests that complex diseases can be largely attributable to a moderate number of common variants, each of which explains partial risk among a population [3]. According to the CDCV hypothesis, the genetic cause is considered to be either a large number of small-effect common variants across the entire allele frequency spectrum, which is also called “the infinitesimal genetic model” [4], or some combination of genotypic, environmental, and epigenetic interactions, known as “the broad sense heritability model” [5].

In the broad sense heritability model, there is a focus on two types of interactions in the quantitative research, which are the genotype-by-genotype interactions, also known as epistasis, and genotype-by-environment interactions. The genotype-by-genotype interactions consider that the effect of one genetic variation is conditional on genotypes at one or more other unlinked loci, while the genotype-by-environment interactions consider that the effect of one genetic variation is conditional on environmental factors, such as behaviors and temperature [3].

Along with the growing evidence of genotype-by-genotype interactions being important contributors to genetic variations in complex human diseases, there are many different formulations in modeling both types of interactions [6]. Some heuristic, learning-based, and Bayesian-based approaches are also proposed, especially for addressing nonlinear interactions and high-dimensional data. This type of approach, to our knowledge, includes machine learning approaches (e.g., neural networks [7, 8]), data-mining approaches (e.g., pattern mining [9]), and regression-based approaches (e.g., classification and regression trees (CARTs), pattern-based logistic regression [10], and logic regression). Logic regression (LR) is one of the approaches for finding multiple interactions and has been successfully applied on several datasets [6, 11–17]. A series of logic regression-based approaches have been developed and are reported to outperform other existing approaches [12–15, 17].

Genetic studies now generate SNP data with thousands or millions of variants from more than ten thousand sampled individuals. A main deficiency of existing LR-based approaches is that these approaches are not efficient enough to handle large-scale data. These approaches often suffer from slow convergence when finding the optimal solutions in a very large solution space. Because of the design of the logic tree (LT, the basic computational unit in logic regression), the size of the solution space of the logic trees increases factorially when the number of SNPs becomes larger. A way of speeding up the logic regression is to design a better regression algorithm. The greedy strategy [11], the simulated annealing algorithm [12, 13], and the bootstrap strategy [14, 15] have been successfully applied in different scenarios.

Motivated by previous studies, in this paper, a novel regression algorithm on the logic regression framework is described. This new algorithm incorporates fish-swarm optimization [18], which is a widely used particle swarm algorithm that is based on swarm intelligence. The basic idea of the fish-swarm algorithm is to introduce a school of fish, which are implemented by threads in the computation. Each fish (fish agent) holds a logic tree and explores the solution space according to a set of preset individual behaviors and swarm behaviors. To speed up the convergence of the search process, the new algorithm also improves the behaviors by introducing selection probability distributions. These probability distributions lead to the selection of more suitable behaviors in the search.

## 2. Background and Related Studies

### 2.1. Basic Logic Regression Model

Logic regression (LR), which was first proposed in [11], attempts to identify a set of Boolean combinations (interactions) among candidate variables (SNPs) for the prediction of a case-control phenotype. A Boolean combination involves interacting SNPs and logic interactions among them. The basic logic regression [11] attempts to find a single Boolean expression that best “explains” the given genotypes behind the observed phenotype. One “explanation” means that the phenotype value predicted by the Boolean expression on a genotype is the same as that of the corresponding phenotype of this genotype. The expression with the highest number of explanations is the output of the regression process. The number of explanations of an expression is also called the score of this expression.

**Figure 1:**
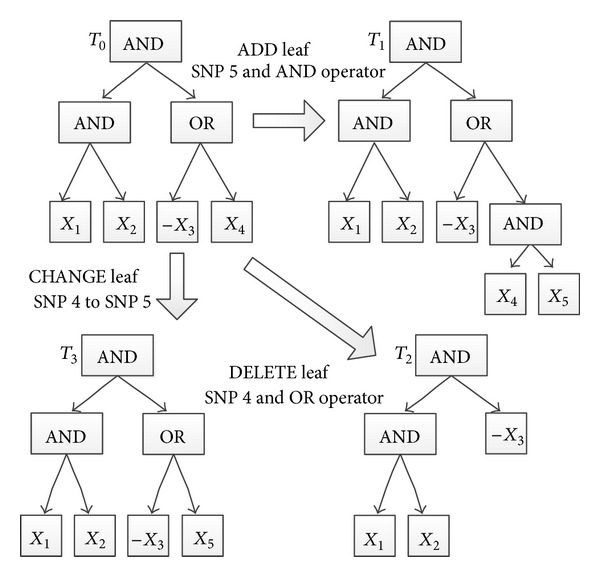
Logic tree representation of *X*_1_∧*X*_2_∧(¬*X*_3_∨*X*_4_) and three permissible moves for logic trees. Starting tree, *T*_0_, is at top left.

Because of the combinatorial explosion of potential Boolean combinations, the logic tree (LT) model is suggested to represent a Boolean expression, where each leaf of a logic tree corresponds to a SNP site, while the internal nodes are associated with logical operators (e.g., AND or OR). A greedy strategy and a simulated annealing algorithm are designed separately to search for a better logic tree that fits the given genotype-phenotype dataset better. Note that every Boolean expression can be represented as a logic tree; see Figure 1. The logic regression is considered to be an exact approach in association studies.

The process of seeking a better logic tree is operated by changing the components or modifying the topology of the current logic tree. In basic logic regression approaches, three tree operations are suggested: add, delete, and change; see Figure 1. The add operation is to add a SNP (or its negation) with a specific operator (AND or OR) to the current logic tree. The delete operation removes a SNP and its parent internal node, the operator, from the current LT. The change operation updates the current LT by changing a SNP (or operator) to a different SNP (resp., operator). Without loss of generality, considering a set of LTs that together influence the phenotype, the basic model of the logic regression is as follows [11]:

(1) $$\begin{array}{c} g\left(E\left(P\right)\right)=\beta_{0}+\sum_{i=1}^{k}\beta_{i}L_{i}, \end{array}$$

where each *L*_*i*_ represents a logic tree among the set of *k* logic trees; these LTs jointly affect the case-control phenotype *P* ∈ {0,1}. The total number of SNPs involved in all of the logic trees is defined as the logic tree model size *s*. Here, *β* are a series of unknown regression parameters, each for a specific logic tree. *E*(*P*)∈[0,1] is the expectation of the phenotype. An invertible link function, *g*, is introduced to map the real-valued predictor *E*(*P*) onto the range {0,1}. According to the existing logic regression-based approaches [11–13], we also adopt the same “sigmoid” function: *g*(*η*) = 1/(1 + exp⁡⁡(−*η*)).

However, many Boolean expressions can fit equally or almost equally well, and there are no universal algorithms to reduce the Boolean expressions. Furthermore, the best Boolean expression may be an overfitted expression rather than the true one. This situation occurs more frequently due to noisy data [12]. To overcome these weaknesses, a Monte Carlo logic regression (MCLR) approach is designed [12], which partly incorporates Bayesian model selection techniques and reports a group of plausible Boolean expressions for further investigation. Different statistical tests are applied on the Boolean expressions, and different interesting features are identified. In the MCLR approach, the model size and the number of models with the same size are treated as random variables following a geometric prior distribution. The prior for a model *M* of size *s* is given by

(2) $$\begin{array}{c} P\left(M\right)=P\left(s\right)P\left(M\;|\;s\right)=\theta \left(1-\alpha \right)\times \alpha^{s}\times \frac{1}{s_{N}}, \end{array}$$

where *s*_*N*_ is the number of models with size *s*. The parameter *α* ∈ (0,1) in the prior is used to penalize large models. The larger a best model is, the more likely it can be achieved by chance. It is reported that the penalty parameter *α* somehow controls the overfitting issue [12]. However, *α* and another model parameter, the maximum number of logic trees *k*, must be predetermined in MCLR. In practice, these two parameters are often suggested by experts on the applications. In another logic regression-based approach, full Bayesian logic regression (FBLR) [13], these parameters are assigned prior distributions and are estimated according to the priors and the posteriors. A more recent logic regression-based approach, LogicFS, suggests that the rank of SNPs and their interactions might be more useful than the combinations themselves. LogicFS serves as the first approach to ranking the interactions of SNPs, by computing the importance measures of the single SNPs, pairwise SNPs, triplets, quadruplets, and so on [14, 15].

### 2.2. Basic Fish-Swarm Optimization Framework

Fish-swarm optimization (artificial fish-swarm algorithm, AFSA) is a swarm optimization framework, which was first proposed by Li and others in [18, 19]. AFSA is a natural computing algorithm, which models some social behaviors of a school of foraging fish. A school of fish is a self-organized group, where each fish has no knowledge about the whole group and environment. Rather than being controlled by a leader, a fish moves around its colony via exchanging information with its adjacent colony members and applying a series of self-organizer behaviors. To model a fish colony and such behaviors, an AFSA is a distributed optimization framework, which consists of a set of fish agents. The environment of an AFSA is the solution space of a particular optimization problem, while a location of a fish agent in this environment corresponds to a solution in the solution space. Each fish agent imitates those social behaviors as a fish. A fish may have many social behaviors; however, in the fish-swarm optimization framework, three major behaviors are considered: preying, following, and swarming.

Preying is a basic biological behavior which describes how a fish tends to eat. For example, a fish perceives a concentration of food in the environment; preying behavior is to determine the movement and the tendency to achieve the concentration position. In an AFSA, the concentration of food in the environment indicates a solution that is better than the current solution where the fish agent is located. The preying behavior in an AFSA illustrates how to reach the better solution from the current one. When a single fish or several fish find the concentration, its adjacent members can trail this/these fish, and thus the swarm will reach the food more quickly. This process is called following. In an AFSA, the following behavior is imitated by comparing solutions among different fish agents. Obviously, following significantly benefits the convergence speed. To enable the following behavior, the fish must assemble the group to guarantee the existence of the colony and neighborhood relationships. On the other hand, any pair of fish cannot get too close because of the limitation of food. Thus, the swarming behavior assembles the fish but prevents them from being too dense. This behavior is very meaningful in AFSA because it prevents the fish swarm from dropping into local optima.

As an optimization framework, different behaviors may be considered and implemented for different problems and solution spaces. Overall, AFSA is suggested as one of the best swarm intelligence optimization methods due to its high convergence speed, flexibility, fault tolerance, and many other advantages [20]. For more details on fish-swarm optimization, Neshat and others wrote a comprehensive review in [20].

### 2.3. Problem Statement

Suppose that we are given a set of *M* sampled individuals. We use a binary vector *P* to represent the phenotype of these *M* individuals. For any individual *i*, *P*_*i*_ = 1 if *i* is a case (affected by the phenotype) and *P*_*i*_ = 0 if *i* is a control (not affected by the phenotype). Let *h*_*i*_ represent a SNP genotype of individual *i* with a set of *N* sites. When we consider the “recessive-set” genetic model, we assume that this SNP genotype is a binary vector that shows the allele types at all sites (which are assumed to contain two alleles each). For the “recessive” genetic model, the logic regression framework can also handle diploid genotypes by encoding each site into a 2-bit binary variable, which is the same as all existing LR-based approaches [11–13]. Thus, we have a binary matrix with *M* rows (each for one of the *M* haplotypes) and *N* + 1 columns, where the first *N* columns correspond to the *N* SNPs and the last column corresponds to the phenotype. Let *S*_*j*_ denote the *j*th SNP. *s*_*i*,*j*_ denotes the allelic value of individual *i* at site *j*. We assume that *s*_*i*,*j*_ = 0 if the SNP *j* of individual *i* shows a wild-type allele, and *s*_*i*,*j*_ = 1 if the SNP *j* of individual *i* shows a mutation. The goal of our problem is to find the interaction(s) among the SNPs (a subset of the given SNPs) that might explain the phenotype better.

## 3. Methods

Our new method is the fish-swarm logic regression algorithm. The main motivation for developing this algorithm is to conduct a more efficient and accurate regression process and to extend the algorithm to a parallel framework. To perform initializations, we first generate *F* fish (fish agents) and *F* initial logic trees. Each fish agent holds a logic tree, separately. The details of initialization are described in Section 3.1. This school of fish agents are seeking better solutions iteratively. In each iteration, fish agents communicate with each other to identify the best (or one of the best) logic tree(s) among all of the logic trees that are held by the school, according to the scores. Then, each fish agent chooses one of the preset behaviors, which are described in Section 3.2, by comparing the current logic tree with the best logic tree. After updating the current logic tree, the new logic tree is accepted to replace the current one by criteria with different conditions. In contrast to most of the existing logic regression-based approaches, the total number of iterations is no longer preset, and our algorithm will terminate when the best logic tree converges.

Our approach takes advantage of swarm optimizations. By incorporating a swarm framework, the algorithm searches the solution space from multiple start points (different logic trees) instead of continuing to apply modifications on one logic tree. Thus, it is obvious that we have a higher probability of converging into local optimum(s) or global optimum(s), and thus, this framework speeds up the previous search process. In particular, we use the “fish agent” framework rather than other swarm intelligence frameworks because of the high similarity between the mechanism of a fish swarm and the genotype-phenotype association problem. In a natural scenario with a school of fish, a fish forages independently in a small space around it, while it also might follow other fish that could lead to a space that has more food. However, each fish always keeps a distance from the other fish, to control the school density. This arrangement is one of the major differences of the fish swarm from the other swarm algorithms. Intuitively, we would like to prevent logic trees from gathering together, because if they do so, then the algorithm might actually perform similar to the algorithm that has only one logic tree performing, and it could fall into a local optimum rapidly. Moreover, as mentioned before, selecting only the best logic tree is not sufficient; the mechanism of the fish swarm fits well with the problem and the requirements.

### 3.1. Logic Tree Space

The fish-swarm algorithm models the natural environment and animal behaviors; however, we cannot blindly or mechanically copy this framework because of two reasons: (1) the solution space that comprises the logic trees is significantly different from the 3D space (the natural environment), and (2) behaviors in the natural environment are not able to directly apply to the logic tree space.

We modify the fish agent framework to fit this specific problem. Suppose that we have generated *F* fish agents, and this school will explore the solution space then. The first issue of building up the framework is whether we could map the solution space to the “environment.” An associated problem is how to describe the differences between the logic trees for the communications among fish agents. In a real-world scenario, the environment is a three-dimensional space, and the space coordinates are communicated. The solution space, on the other hand, comprises all of the possible logic trees, where the logic trees could have multiple dimensions. For example, two different logic trees not only might bring a different number of SNPs but also might be built up by different topologies or different logic operators on the internal nodes. Here, we propose a practical way to map logic trees in a four-dimensional space and measure the differences among them, which is enlightened by a full Bayesian framework on logic regression in [13].

First, in the logic regression framework, there are three major unknown parameters: the number of logic trees in one regression model *n*_*l*_, the size of the regression model *s*, which is equal to the number of SNPs involved in the model, and the SNPs involved in each logic tree $\vec{q}$. The priors of these parameters are written in a factorized form:

(3) $$\begin{array}{c} p\left(\text{Model}\right)=p\left(n_{l}\right)\cdot p\left(s\right)\cdot \prod_{i=1}^{n_{l}}p\left(s_{i}\right)\cdot p\left({\vec{q}}_{i}\;|\;s_{i}\right), \end{array}$$

where *s*_*i*_ is the size of the logic tree *i* in the regression model (1 ≤ *i* ≤ *n*_*l*_) and vector ${\vec{q}}_{i}$ is a binary indicator for logic tree *i*, in which *q*_*i*,*j*_ = 1 denotes that the SNP *j* is selected as a leaf of logic tree *i*. In other words, *s*_*i*_ is also equal to the number of 1*s* in ${\vec{q}}_{i}$.

We assume that the size of every logic tree has a prior distribution of *p*(*s*_*i*_) = *U*(1,…, *s*_max⁡_), where *U*(·) represents a uniform distribution and *s*_max⁡_ is the maximum size of a logic tree that can be preset. *p*(*n*_*l*_) is also assigned a prior distribution of *p*(*n*_*l*_) = *U*(1,…, *n*), which indicates the lack of knowledge of the number of logic trees. In a specific case, both the prior distributions of *p*(*s*_*i*_) and *p*(*n*_*l*_) can be preset to incorporate more prior knowledge.

A logic tree not only makes up the SNPs but also connects with the logic operators. The logic operators describe complex interactions among the SNPs. Different SNPs could have different functions, including “causal,” “neutral,” and “protective.” The causal variants increase the risk of cases, while the protective variants decrease the risks. The neutral variants are considered to be independent of the phenotype. For the “additive” genetic model, the “AND” operators are adopted to connect the causal SNPs. For the “dominant” genetic model, the causal SNPs are connected by “OR” operators. If we split a logic tree into two sublogic trees (sub-LTs) at an “OR” operator, according to the genetic model, each new sub-LT affects the phenotype independently, which is the same as the original logic tree. In other words, these two new sub-LTs still contain the same information as the original logic tree. This arrangement implies that splitting the logic tree at an “OR” operator will not cause information loss. Thus, if we split a logic tree at the “OR” operators recursively, we obtain a forest (a set of logic trees) that comprises the sub-LTs with only the “AND” operators.

We highlight the split process for two reasons.

- The sub-LTs in the forest contain only “AND” operator(s), and thus the topologies of these sub-LTs are no longer considerable because of the commutative law. The differences between any two sub-LTs, sub-LT *i* and sub-LT *j*, can be represented by the differences between vector ${\vec{q}}_{i}$ and ${\vec{q}}_{j}$.
- The forest represents all of the information of an original logic tree, and thus the differences between two logic trees are computable by measuring the forests that are derived from them. For example, the number of “OR” operators in a logic tree is equal to the number of sub-LTs in the forest.

In summary, we define a three-dimensional hyperspace as follows: the first dimension, the scalar *n*, indicates the number of sub-LTs that can be derived from a logic tree; the second dimension, vector $\vec{s}$, indicates the size of each sub-LT; and the third dimension, scalar *Y*, indicates the score of the original logic tree. For a simpler version, the second dimension can be replaced by a scalar *s* of the size of the original logic tree. According to our experience, the accuracy of this simplified version is roughly the same as the original one.

### 3.2. Behaviors of Fish Agents

When we have the search space, fish agents conduct behaviors that search the solution space simultaneously. Thus, to define the behaviors that regulate the search strategy is another important part of a swarm algorithm. Behaviors are often dependent on the solution space that they work on. For the specific logic tree space, note that we have a collapsing solution space from the set of all possible logic trees rather than a bijective solution space. For example, one point in the logic tree space could correspond to multiple logic trees. This correspondence occurs because of the complexity of both the tree topology and the logic operators. For a bijective solution space, defining the swarm behaviors is adequate in most cases; however, in the logic tree space, the fish agent should harbor necessary behaviors itself, in addition to the swarm behaviors, to update the logic tree that it holds even when it keeps the location in the space. In this section, we will describe the behaviors for a fish agent, while in Section 3.3, we will define the behaviors of the fish swarm. For a fish agent *f*_*i*_, we define four fish agent behaviors that allow it to alter its current logic tree to a new logic tree. These four behaviors are the following.

1. ADD SNP: select a SNP and add it to the LT.
2. DEL SNP: select a SNP on the LT and remove the SNP from all of the sub-LTs.
3. ALT SNP: select a SNP on the LT and alter the SNP by another SNP.
4. ALT OPT: select an operator on the LT and alter the operator by the opposite operator.

The probability distribution of choosing a behavior affects the preferences of the behaviors. The simplest way is to adopt a uniform distribution; for example, each behavior has the same probability, 25% of them being chosen. However, to accelerate the convergence, it is better to reflect preferences among the behaviors. Suppose that, after one iteration, the fish agent that holds the logic tree with the highest score is announced. Let this fish agent be *f*_best_. Intuitively, when the *f*_best_ is announced, the difference in the sizes between fish agents *f*_*i*_ and *f*_best_ can be measured by $||{\vec{s}}_{i}-{\vec{s}}_{j}||$. Thus, we adopt a normal distribution to obtain the chosen probability of each behavior, where the probability density function subjected to $\vec{s}$ is

(4) $$\begin{array}{c} f\left(s\right)=\frac{1}{\sqrt{2\pi \sigma^{2}}}e^{-{\left(s-s_{\text{best}}\right)}^{2}/2\sigma^{2}}, \end{array}$$

where the mean of this normal distribution is set to *s*_best_, which is the size of *μ* = *f*_best_, and the variance of this normal distribution is set to *σ* ^2^ = (1/*m*)∑_*i*=1_^*m*^(*s*_*i*_−*s*_best_)^2^.

Furthermore, for a specific SNP, we should also consider the probability that this SNP will be chosen. We obtain the probability distribution of selecting a SNP by measuring the importance of each SNP. The measurement of importance is a statistic [14, 15]. Intuitively, an important SNP is assumed to be the SNP that occurs most frequently. However, although some SNPs either may be explanatory for a small subset of cases and controls or could be actually very important for the correct prediction of some of the phenotypes, such SNPs are considered to be unimportant [14]. Every several iterations, the algorithm updates the importance of all of the SNPs and generates a new probability distribution.

To compute the importance, each fish agent records the correctly classified out-of-bag (OOB) observations. Let *P*_*i*_(*x*_*j*_) represent the probability of SNP *j* being added to a fish agent *f*_*i*_. The importance of *j* is

(5) $$\begin{array}{c} V\left(j\right)=\frac{1}{F}\sum_{k=1}^{F}\left(N_{k}-N_{k}^{-j}\right), \end{array}$$

where *F* is the number of fish agents and *N*_*k*_ is the number of correctly classified OOB observations of fish agent *f*_*k*_. This measurement is much more robust than the previous measurement based on *β* distributions [17]. Note that each fish agent applies a bootstrap sampling on the given data *D* instead of working on the whole *D*, because it should not be computed on the same data on which the classification rule has been trained but instead is computed on independent data that contains new observations [14].

Suppose there is an index vector *S*_*f*_*i*__ for fish *f*_*i*_ that comprises the SNPs in *f*_*i*_ and an index vector *S*_*f*_*j*__ of fish *f*_*j*_ that comprises the SNPs in *f*_*j*_; then, the distance between *f*_*i*_ and *f*_*j*_ in the solution space can be computed by the distance between *s*_*f*_*i*__ and *s*_*f*_*j*__ as follows:

(6) $$\begin{array}{c} d\left(S_{f_{i}},S_{f_{j}}\right)=\left|\sum_{f_{i}}V\left(i\right)-\sum_{f_{j}}V\left(j\right)\right|. \end{array}$$

### 3.3. Behaviors of the Fish Swarm

Here, we continue to introduce the swarm behaviors. The behaviors of fish *f*_*i*_ are listed as follows.

*(1) HOLD*. If *f*_*i*_ is *f*_best_, then only “HOLD” behavior is allowed. At this time, *f*_best_ does nothing but retains the current best logic tree. Otherwise, *f*_*i*_ will select one of the following three behaviors.

*(2) RANDOM*. If *f*_*i*_ selects “RANDOM” behavior, it searches the space randomly. All of the operations are selected with the same probability 1/4. 

*(3) FOLLOW*. If *f*_*i*_ selects “FOLLOW” behavior, then it will follow the *f*_best_, which indicates that *f*_*i*_ will attempt to

- have the same size as *f*_best_ or
- have the same SNPs as *f*_best_.

To achieve this goal, *f*_*i*_ checks the following *s* value.

**Figure 2:**
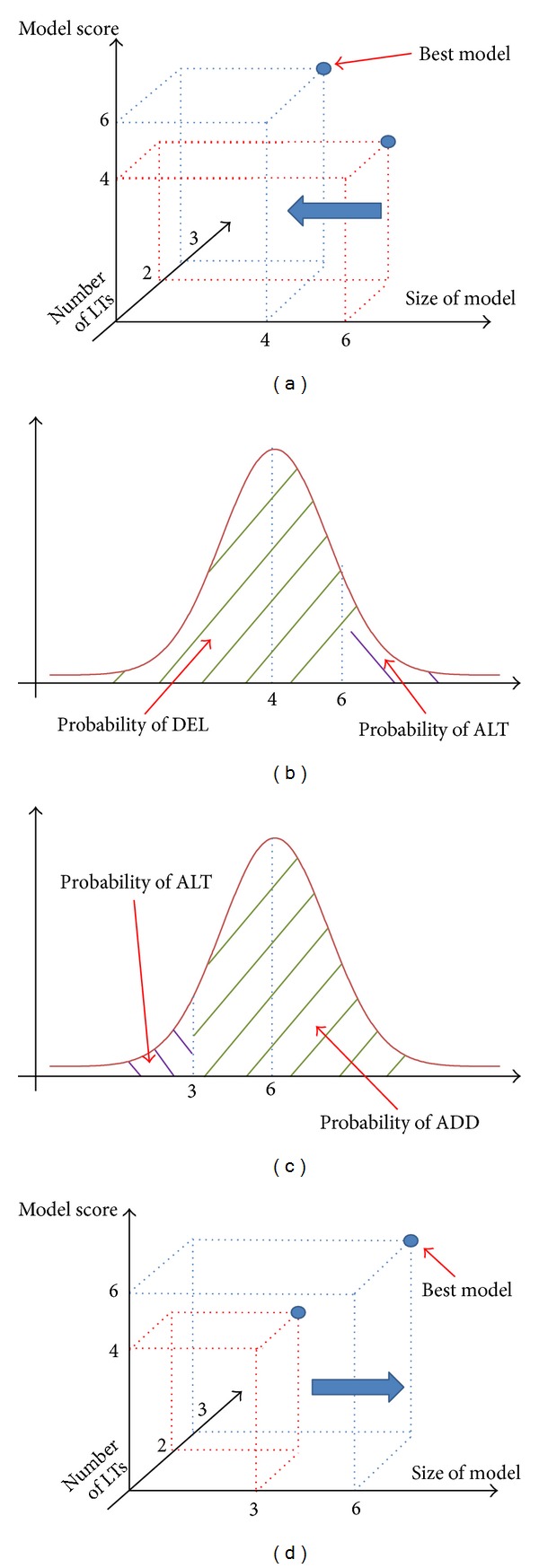
“FOLLOW” behavior is illustrated. When *s*_*i*_ > *s*_best_ (*s*_best_ is equal to 4, and *s*_*i*_ is equal to 6; shown at (a)), the probability of “DEL” operations (shown as the green shadow) is larger than the probability of “ALT” operations (shown as the purple shadow). When *s*_*i*_ < *s*_best_ (*s*_best_ is equal to 6, and *s*_*i*_ is equal to 3; shown at (d)), the probability of “ADD” operations (shown as the green shadow) is larger than the probability of “ALT” operations (shown as the purple shadow).

- If *s*_*i*_ > *s*_best_, then *f*_*i*_ holds more SNPs than *f*_best_. We force that *f*_*i*_ can only choose “DEL” operation or two “ALT” operations. *f*_*i*_ may select “DEL” operations with probability *f*(*s*_*i*_) (delete a SNP *j* with probability 1 − *P*_*i*_(*j*)) and “ALT” operations with probability 1 − *f*(*s*_*i*_) (select a SNP *j* with probability 1 − *P*_*i*_(*j*) and replace it by another SNP *j*′ selected with probability 1 − *P*_*i*_(*j*′)); see Figure 2.
- If *s*_*i*_ < *s*_best_, then *f*_*i*_ holds fewer SNPs than *f*_best_. We force that *f*_*i*_ can only choose “ADD” operation or two “ALT” operations. Then, *f*_*i*_ may select “ADD” operations with probability 1 − *f*(*s*_*i*_) (add a SNP *j* on a LT or add *j* as a new LT with probability *P*_*i*_(*j*)) and “ALT” operations with probability *f*(*s*_*i*_) (select a SNP *j* with probability 1 − *P*_*i*_(*j*) and replace it by another SNP *s*_*j*′_ selected with probability 1 − *P*_*i*_(*j*)); see Figure 2.
- If *s*_*i*_ = *s*_best_, then *f*_*i*_ holds the same number of SNPs as *f*_best_. We force *f*_*i*_ to choose “ALT” with a probability of 1 (select a SNP *j* with a probability of 1 − *P*_*i*_(*j*) and replace it by another SNP *j*′ that is selected with probability 1 − *P*_*i*_(*j*′)).

*(4) KPDIST*. If *f*_*i*_ selects “KPDIST” behavior, then it will keep a distance from *f*_best_, which indicates that *f*_*i*_ will attempt to

- change to a different size from *f*_best_ or
- select different SNPs with *f*_best_.

To achieve this goal, *f*_*i*_ checks the *s* value.

- If *s*_*i*_ > *s*_best_, then we force that *f*_*i*_ can choose only “ADD” operation, with probability of 1 − *f*(*s*_*i*_), and choose two “ALT” operations with probability *f*(*s*_*i*_).
- If *s*_*i*_ < *s*_best_, then we force that *f*_*i*_ can only choose “ALT” operations and “DEL” operation with probability *f*(*s*_*i*_) and 1 − *f*(*s*_*i*_), respectively.
- If  *s*_*i*_ = *s*_best_, then we require that *f*_*i*_ can only choose “ADD” and “DEL” operations with the same probability of 1/2.

After applying a series of behaviors, each fish agent holds a new logic tree. If the new logic tree obtains a higher score than the previous logic tree, then the new logic tree explains more genotypes; next, this new logic tree is accepted and replaces the previous one. Otherwise, the new logic tree is rejected with a probability of *Q* [12]:

(7) $$\begin{array}{c} Q=\min\left\{1,\frac{v^{k/2}{\left|{\hat{V}}^{*}\right|}^{1/2}}{v^{k^{*}/2}{\left|\hat{V}\right|}^{1/2}}\exp\left(\frac{a}{a^{*}}\right)\right\}, \end{array}$$

where *v* determines the prior variance, $\left|\hat{V}\right|$ is the determinant of the posterior variance covariance matrix, and *a* is an error term to measure the fitness to the data *Y*, where a *gamma function* is applied as $a=\Omega^{\prime }\Omega -{\hat{\beta }}^{\prime }{\hat{V}}^{-1}\hat{\beta }$. *Ω* is the covariate matrix within the regression framework, *β* is the associated vector of coefficients, and $\hat{\beta }$ is the maximum a posteriori estimate of *β* which is found by Newton's method. The superscript ∗ refers to the parameters of the proposed updated model, and other parameters are denoted for those of *f*_best_. This acceptance probability *Q* in an equation is modified to check the proximity of the current solution (LT *M* ^0^) with the global optimal (LT M*).

### 3.4. Accepting the Candidate Models

 Each fish agent could store a local optimal logic tree during the search process, while the whole swarm always announces the current best logic tree (the global optimal). After several iterations, the reversible jump method is implemented. In other words, the acceptance probability of a newly proposed logic tree could decrease, but it might be closer to the best LT in the current iteration.

In addition, we consider a stepwise regression process. The stepwise regression eliminates insignificant SNPs iteratively and drops them off. The stepwise mechanism checks the active SNPs (SNPs not removed) every *I*_max⁡_ iteration and determines whether a SNP should be removed or not, according to the results of an *F* statistic. At the same time, the level of significance *F*_out_ under the *F* statistic is introduced for determining whether an independent SNP should be removed or not. To achieve this goal, we calculate the total sum of squares (TSS) after dropping SNP *i*  (*i* = 1,2,…, *n*):

(8) $$\begin{array}{c} \text{TSS}=\text{RSS}{\left|{}_{k}+\text{ESS}\right|}_{k}, \end{array}$$

where *k* = 1,…, *n* and *k* ≠ *i*. RSS is the sum of squares due to the regression, and ESS is the sum of squares due to the random errors or the residuals. We drop SNP *i* if the *F* statistic of SNP *iF*_*i*_ ≤ *F*_out_, where *F*_out_ is a preset threshold.

Finally, when *f*_best_ does not change in *B* iterations (the threshold *B* is preset), the regression algorithm terminates and outputs all of the logic trees that are held by all of the fish in the school.

## 4. Results and Discussion

We first apply our fish-swarm logic regression (FSLR) approach on a real screening dataset and then apply it on a series of simulated datasets under different configurations to test the performance of our approach compared to other logic regression-based approaches. The software tool, FSLR, is available at http://www.engr.uconn.edu/~jiw09003/.

Three existing LR-based approaches are compared, which are Monte Carlo logic regression (MCLR) [12], full Bayesian logic regression (FBLR) [13], and the SNP(s) importance measurement approach (LogicFS) [14]. The software package attached to MCLR is LogicReg, the software package for FBLR is SCRIME, and for the SNP importance measurement approach, LogicFS is the name of the software package. We adopt two groups of parameters in MCLR: (1) *α* = 1/2 with the total number of LTs *K* = 2, and (2) $\alpha =1/\sqrt{2}$ with *K* = 3, which are suggested in [12, 13]. Both MCLR and FBLR are preset to run 100,000 iterations with an additional 10,000 burn-in iterations in all of the experiments, as suggested.

### 4.1. Fish Swarm on Real Mutation Screening Data

The real dataset is from our own study, which focuses on the genetic association between the dopamine receptor D1 (DRD1) gene polymorphisms and the risk of opioid dependence. Seven possible functional single nucleotide polymorphisms, rs4867798, rs1799914, rs686, rs4532, rs5326, rs10063995, and rs10078866, in the regulatory or coding regions of DRD1 were identified by DNA sequencing in 20 heroin addicts and were further genotyped in 425 heroin addicts and 514 healthy controls.

Several genes that encode dopamine receptors have been confirmed to be associated with a risk of heroin addiction. Our previous studies [21, 22] as well as some studies from other labs [23–25] indicate that TaqI RFLP in the dopamine receptor D2 gene (DRD2) and −521 C/T in the Dopamine receptor D4 gene (DRD4) modulate the predisposition to heroin dependence. Several noncoding but potentially functional polymorphisms in flanking or untranslated regions of DRD1 have been identified, such as rs686 (+1403 T/C), rs4532 (−48 A/G), and rs5326 (−94 G/A). The rs686 polymorphism has been proven to affect the expression levels of DRD1 and might influence the level of DRD1 stimulation in PFC [26, 27]. Two types of drug dependence, nicotine [26] and alcohol [28], and addictive behavior (pathological gambling) [29] have both been shown to be associated with DRD1 (rs686, rs4532, and rs265981), which suggests that there are common effects of DRD1 on the susceptibility to addiction.

We applied our approach, FSLR, on this dataset. When considering the homozygote mutations, the logic regression model reports the highest score, which is 516 (among 939 individuals). rs4532 is the SNP with the highest importance. Two interactions, rs4532-rs686 and rs10078866-rs4532, are accepted much more than other interactions. When considering both the homozygote and the heterozygote mutations, two interactions, rs4532-rs1799914 and rs1799914-rs686, are accepted much more than the others, with the highest score being 518. These results, which are for candidate associations, are supported by clinical knowledge.

### 4.2. Simulated Data Preparation

For each simulation configuration, we generate 100 datasets. All of the datasets are generated by the *ms*-series simulator [30]. The *ms*-series is widely used for generating haplotypes/genotypes with preset parameters, for example, the mutation rate and recombination rate. For each dataset, we first use *ms* to generate 40,000 haplotypes with the same number of segregating sites equal to 1000. The neutral mutation rate is equal to 10^−7^, and the crossover probability between adjacent base pairs is equal to 10^−7^ per generation. Then, we randomly pick up a specific set of preset causal sites and generate a Boolean expression among them. According to the Boolean expression, we compute the phenotype of each haplotype: if the output of the expression is equal to 1, then the haplotype is a case; otherwise, the haplotype is a control. Note that complex traits are often affected by multiple factors. So we define the level of risk as equal to the probability of the phenotype being the same as the output of the Boolean expression. In other words, an individual that has mutations on causal sites has a higher probability of being a case, rather than having 100% chance of being a case. This probability is equal to the risk. Finally, we randomly sample 1,000 haplotypes from cases and 1,000 haplotypes from controls to make up one dataset. Moreover, datasets always have some errors. To get closer to the real datasets, we add noise (errors) on the simulated datasets. For the generated haplotypes, we define the level of noise as equal to the probability of randomly altering an allelic value from wild type to mutation or from mutation to wild type. We add the noise on the haplotypes randomly after the 2,000 haplotypes are sampled.

In the following sections, we will present the comparison results on three aspects: (1) the accuracy of each approach (measured by the type I and type II error rates), (2) the performance under different levels of risk and different levels of noise, and (3) the running time. To ensure confidence in the results, we conducted 100 repeats for each configuration used in the comparison.

### 4.3. Accuracy for the Different Numbers of Causal SNPs

We first compared the accuracy. The accuracy is measured by the type I error rate and the type II error rate, separately. The type I error rate is computed as the percentage of missed causal sites divided by the number of selected SNPs, while the type II error rate is computed as the percentage of wrong selections of noncausal SNPs among all of the SNPs involved in a regression model. The given datasets always have 1000 sites for every genotype, but the number of causal sites varies from 10 to 100 among the 1000 sites. In other words, the proportions of causal variants decrease from 1% to 10%.

 The results of the type I and type II error rates are compared in Table 1. The column “Causal” shows the number of causal sites. In most of the configurations, our approach, FSLR, has lower type I error rates than FBLR and LogicFS. For example, when the number of causal sites is larger than 30, FSLR always has a decrease of 1-2% in the type I error rate and has an ~10% lower type II error rate than the other two methods. Note that MCLR always has low (approximately 1%) type I error rates but obviously high (almost 100%) type II error rates. To investigate this phenomenon, we also recorded the average number of successfully identified preset causal sites by each approach, for which the results are shown in Table 2. MCLR appears to be a more aggressive approach, which reports more candidate causal sites than the other three methods. The remaining approaches appear to be more conservative, because their regression models are smaller than those of MCLR. However, according to Table 2, MCLR only reports 1 or 2 preset causal sites, although it has lower type I error rates, while other approaches are more practical and find many more.

### 4.4. Accuracy at Different Noise Levels

We also compared the accuracy under different levels of risk and different levels of noise. All of the datasets applied in this group of experiments have a total of 10 preset causal sites among the 1000 sites. We first varied the levels of risk from 5% to 15%; then, we varied the levels of noise on the haplotypes from 1% to 3%.

The results are compared in Tables 3 and 4. According to these results, we can conclude that the performance of FSLR, FBLR, and LogicFS is weakened either by the risk or by the noise. However, FSLR can identify more causal sites than FBLR and LogicFS. For example, FSLR successfully reports approximately 20 preset causal sites, while FBLR finds only ~11 and LogicFS only identifies 13 approximately. The reason that FSLR has higher type I error rates is that the regression models (the number of candidate causal sites) reported by FSLR are smaller than those of the other two methods. Thus, although FSLR misses fewer preset causal sites, the type I error rates are still higher. On the other hand, MCLR is not affected significantly by these, but the original performance of MCLR might not be good enough.

### 4.5. Comparisons of the Running Time

In addition, we compare the running time among FSLR, MCLR, FBLR, and LogicFS. We record the average running time on 1000 repeats. Because both MCLR and FBLR rely on the Monte Carlo Markov chain (MCMC) to seek a better regression model, the number of iterations of MCMC might dominate the running time. Following the suggestions in the papers, we preset 100,000 iterations with an additional 10,000 burn-in iterations. LogicFS is preset by 20 iterations with bootstrap sampling. FSLR is applied on a cluster with 12 laptops. The collections of running time are shown in Table 5. Intuitively, FSLR runs faster than MCLR and FBLR, while it has a similar speed to LogicFS.

## 5. Conclusions

In this paper, we present a novel logic regression-based approach, fish-swarm logic regression (FSLR), to detect the interacting SNPs that are associated with a phenotype. We designed a new regression algorithm, which incorporates the advantages of a swarm framework, to improve both the accuracy and the efficiency of logic regression. In contrast to previous swarm algorithms, in this approach, we design a specific solution space into which all possible logic trees are mapped. Then, two types of behaviors, agent behaviors and swarm behaviors, are suggested to rule the search strategy. A series of simulation experiments are performed to compare the accuracy under different scenarios of three logic regression-based approaches. The running times among the approaches are also collected. Our approach, fish-swarm logic regression, often outperforms other approaches in terms of the accuracy under different simulation configurations, and it has a better running time on parallel frameworks than that of the others.

## Figures and Tables

**Table 1:** Accuracy for different numbers of causal SNPs. The column “Causal” shows the number of casual sites. The type I error rate is the percentage of missed causal sites divided by the number of selected SNPs. The type II error rate is the percentage of wrong selections of noncausal SNPs among all of the SNPs involved in a regression model. For each simulation configuration, the number is computed based on 100 repeats.

Causal	FSLR		MCLR		FBLR		LogicFS	
	Type I	Type II	Type I	Type II	Type I	Type II	Type I	Type II
10	0.65%	65.00%	1.38%	88.30%	0.52%	52.00%	0.63%	63.00%
20	1.38%	69.00%	1.21%	94.75%	1.34%	67.00%	1.47%	73.50%
30	1.75%	58.33%	1.20%	96.13%	2.15%	71.67%	2.21%	73.67%
40	2.53%	63.25%	1.18%	97.30%	3.02%	75.50%	3.22%	80.50%
50	3.72%	69.40%	1.14%	97.64%	4.05%	81.00%	3.98%	79.60%
60	3.80%	63.33%	1.10%	97.90%	4.73%	78.83%	4.90%	81.67%
70	4.62%	66.00%	1.08%	98.17%	5.78%	82.57%	5.82%	83.14%
80	5.40%	67.50%	1.09%	98.48%	6.24%	78.00%	6.58%	82.25%
90	5.38%	59.79%	1.10%	98.91%	7.24%	80.44%	7.67%	85.22%
100	6.44%	64.40%	1.05%	98.40%	7.76%	77.60%	8.47%	84.70%

**Table 2:** Comparisons on identifying preset causal sites. The column “Causal” shows the number of casual sites. A column under the name of an approach shows the average number (among 100 repeats) of successfully identified preset causal sites among the number of casual sites.

Causal	FSLR	MCLR	FBLR	LogicFS
10	3.5	1.23	4.8	3.7
20	6.2	1.05	6.6	5.3
30	12.5	1.16	8.5	7.9
40	14.7	1.08	9.8	7.8
50	12.8	1.18	9.5	10.2
60	22.0	1.26	12.7	11.0
70	23.8	1.28	12.2	11.8
80	26.0	1.22	17.6	14.2
90	36.2	0.98	17.5	13.3
100	35.6	1.60	22.4	15.3

**Table 3:** Accuracy for different numbers of causal SNPs with risks and noise. The level of risk is equal to the probability of the phenotype being the same as the output of the Boolean expression. The level of noise is equal to the probability of randomly altering an allelic value from wild type to mutation or from mutation to wild type. The type I and II error rates are similar. For each simulation configuration, the number is computed based on 100 repeats.

	FSLR		MCLR		FBLR		LogicFS	
	Type I	Type II	Type I	Type II	Type I	Type II	Type I	Type II
Risk								
5%	12.8%	58.80%	1.16%	98.88%	8.70%	73.60%	6.80%	71.80%
10%	12.7%	59.00%	1.12%	98.44%	8.90%	77.60%	6.70%	73.40%
15%	12.3%	59.20%	1.19%	98.92%	8.90%	74.40%	6.60%	73.90%
Noise								
1%	12.5%	59.00%	1.17%	98.56%	8.90%	77.80%	6.70%	73.40%
2%	13.5%	58.00%	1.17%	98.76%	9.00%	81.80%	6.80%	74.60%
3%	14.8%	56.60%	1.08%	99.19%	8.90%	78.40%	7.30%	76.60%

**Table 4:** Comparisons on identifying preset causal sites with risks and noise. A column under the name of an approach shows the average number (among 100 repeats) of successfully identified preset causal sites under the particular level of noise.

Noise	FSLR	MCLR	FBLR	LogicFS
5%	20.6	0.72	11.1	13.3
10%	21.0	0.62	9.1	13.7
15%	21.7	0.50	10.8	11.7

1%	20.6	0.56	13.2	14.1
2%	20.5	1.28	11.2	13.3
3%	19.9	0.54	12.8	12.5

**Table 5:** Comparisons on running time. The running time is measured in seconds.

Causal	FSLR	MCLR	FBLR	LogicFS
10	17.43	56.59	1659	12.23
20	18.48	53.64	1559	12.50
30	18.72	58.37	1603	12.12
40	18.96	57.76	1463	11.88
50	19.45	58.31	1520	12.10
60	19.94	57.72	1418	12.43
70	20.69	59.58	1482	12.11
80	22.49	58.04	1366	12.57
90	24.35	58.54	1466	12.79
100	24.35	59.13	1346	12.65
